# The PinX1/NPM interaction associates with hTERT in early-S phase and facilitates telomerase activation

**DOI:** 10.1186/s13578-019-0306-y

**Published:** 2019-06-13

**Authors:** Sai-Tim Ho, Rui Jin, Derek Hang-Cheong Cheung, Jun-Jian Huang, Pang-Chui Shaw

**Affiliations:** 1 Centre for Protein Science and Crystallography, School of Life Sciences, The Chinese University of Hong Kong, Shatin, N.T, Hong Kong, China; 2 Laboratory of Tumor and Molecular Biology, Beijing Institute of Biotechnology, Beijing, China

**Keywords:** PinX1, Nucleophosmin, Telomerase, Telomere shortening, Cell cycle, Immunofluorescence

## Abstract

**Background:**

Telomere maintenance is an important factor in tumorigenesis. PinX1 is a potent telomerase regulator which also involves in telomerase loading to telomeres. Nucleophosmin (NPM) can partially attenuate PinX1 inhibition of telomerase activity and NPM loading to hTERT requires PinX1. However, the role of the PinX1/NPM interaction in telomerase activity is not fully understood.

**Method:**

The long-term effects of PinX1 and NPM down-regulation on telomere length were investigated by TRF assay. The localization of the PinX1/NPM association and the NPM/PinX1/hTERT complex formation were examined by immunofluorescence studies.

**Results:**

Concurrent long-term down-regulation of PinX1 and NPM led to a substantial decrease in telomere length. The interaction with PinX1 was crucial in NPM localization in the nucleolus during the S phase. PinX1 and NPM associated throughout S phase and the NPM/PinX1/hTERT complex formation peaked during the early-S phase. The PinX1/NPM interaction was shown to localize away from Cajal Bodies at the start of S phase.

**Conclusion:**

PinX1/NPM interaction is important in telomerase regulation during catalysis. NPM is recruited to hTERT by PinX1 and is required in the proposed telomerase modulating unit to activate telomerase when telomere extension occurs during S phase.

**Electronic supplementary material:**

The online version of this article (10.1186/s13578-019-0306-y) contains supplementary material, which is available to authorized users.

## Introduction

Human telomerase only has a low to undetectable level of activity in normal somatic cells, but is highly active in more than 85% of cancers [1, 2]. The enzyme consists of an enzymatic component (hTERT) and a RNA template component (hTR) and is responsible for maintaining the length of telomeres, which are non-coding G-rich DNA (TTAGGG) repeat sequences at the end of the chromosomes [3]. Telomeres are protected by the shelterin complex, which consists of six protein components—RAP1, TIN2, TPP1, TRF1, TRF2, and POT1 [4, 5]. Telomerase and shelterin complex are critical for telomere length maintenance and thus are of interest in cancer biology.

PIN2/TRF1-interacting telomerase inhibitor1 (PinX1) is a telomerase inhibitor that binds to hTERT and hTR [6, 7]. The overexpression of PinX1 would decrease telomerase activity, thus shortening telomeres, and lower cancer cell tumorigenicity [6, 8]. However PinX1 is not solely a negative regulator of telomerase. The silencing of PinX1 would disrupt the association between telomeres and telomerase [9]. In addition, silencing PinX1 expression led to a substantial telomere length shortening and growth inhibition in telomerase-positive human cancer cells [10]. Besides increasing sensitivity to DNA damage in cancer cells, PinX1 was also shown to suppress cell cycle progression [10, 11]. In urothelial carcinoma of the bladder (UCB), it was suggested that PinX1 inhibits UCB cell growth/proliferation by regulating the expression of the key cell cycle genes for p16 and cyclin D1 [12].

The role of PinX1 in tumorigenesis is complicated. Previously, we have discovered nucleophosmin (NPM) as a novel interacting partner of PinX1. NPM binding to hTERT required PinX1 and NPM could attenuate the PinX1 inhibition on telomerase activity [13]. NPM is an abundant nucleolar protein involved in multiple biological processes such as cellular proliferation, ribosome biogenesis, mRNA processing, DNA damage response, and centrosome duplication [14, 15]. It also shuttles between nucleolus and cytoplasm and acts as a molecular chaperone [16, 17]. NPM is often overexpressed in cancers and positively regulates telomerase activity [18].

While hTERT is synthesized in cytoplasm, hTERT distribution is dynamic during the cell cycle. In G1 phase, hTERT is concentrated in nucleoplasmic TERT foci away from Cajal Bodies (CBs) and in the nucleolus while hTR is enriched in CBs. CBs are dynamic subnuclear structures important for telomerase recruitment to telomeres [19, 20]. hTR and hTERT are assembled into catalytically active telomerase and recruited to CBs [21]. Telomerase transiently translocates out of CBs through the interaction between the TEN domain of hTERT with TPP1 and load onto telomeres during S phase [22]. PinX1 acts as a molecular linker between NPM and telomerase for the NPM/hTERT association and both PinX1 and NPM regulate telomerase activity [13]. Hence, the PinX1/NPM interaction and by extension the hTERT/PinX1/NPM association should also occur during the time of telomerase activation for PinX1/NPM to play a part in telomere length homeostasis. PinX1 could recruit the positive telomerase regulator NPM for loading to hTERT to activate telomerase and facilitate catalysis. The association of NPM, PinX1, and hTERT could account for the duality of PinX1 in the context of telomere length maintenance. Hence, a better understanding of the timing of formation and localization of hTERT/PinX1/NPM complex during cell cycle progression is necessary.

In this report, we provide evidence that the hTERT/PinX1/NPM complex formation peaks during early-S phase and thus PinX1/NPM interaction is important in telomerase regulation during catalysis.

## Materials and methods

### Plasmids

For mammalian cell expression, wild type NPM was cloned into pEGFP-C1 (Clontech Laboratories, USA). The site-directed mutagenesis was introduced by over-lapping PCR and all of the NPM mutants were cloned into pEGFP-C1.

### Cell culture and transfection

HepG2, HEK293T, HeLa (ATCC, USA) cells were cultured in Minimal Essential Medium (Gibco, Invitrogen, USA) with 10% fetal calf serum (Gibco, Invitogen, USA) in 37 °C with 5% CO_2_. Plasmid DNA was transfected into cells by Lipofectamine 2000 (Invitrogen, USA) for 24–48 h.

### Transient down-regulation of protein expression

80 pmol of PinX1 siRNA (On-Target Plus SmartPool, Dharmacon, USA; Stealth siRNA, Invitrogen, USA) or NPM siRNA (On-Target Plus SmartPool, Dharmacon, USA; Silencer Select Pre-designed siRNA Product, Ambion, USA) was transfected using Lipofectamine RNAiMAX (Invitrogen, USA) according to the manufacturer’s protocol. The cells were harvested 48 h post-transfection.

### Long-term down-regulated cell lines and TRF assay

10^6^ HepG2 cells were transfected by 80 pmol PinX1 or NPM siRNA with RNAiMAX (Invitrogen, USA) and grown in 6-well plates and reseeded every 3 days. In the following propagation, the cells were transfected by the same siRNA every 9 days. The long-term effect of endogenous RNAi silencing was regularly monitored by RT-PCR assay at time-point before each siRNA transfection. To measure telomere-length during extended culture, harvested cells were pelleted and the extracted genomic DNA were digested with *Hinf*I and *Rsa*1 overnight before electrophoresis and Southern-blotted with the digosin end-labeled (CCCTAA) oligonucleotide probe. The hybridization signals were detected by HRP-conjugated anti-digosin antibodies (Roche, USA) and imaged by the ECL reagent. Telomere lengths were analyzed with Telotool (Göhring et al., Medical University of Vienna, Austria).

### Immunoblots

Cells were washed once with PBS and 500 μl of ice-cold immunoprecipitation (IP) buffer (50 mM Tris pH 7.6, 150 mM NaCl, 1 mM EDTA, 1% Triton X-100, protease inhibitor cocktail) was added to the cells. The cells were harvested and sonicated for 5 sec. The cell lysate was centrifuged at 14,000 rpm for 10 min at 4 °C. The samples were heated at 95 °C for 10 min and then analyzed by western blotting. Briefly, the cell lysates were separated by SDS–polyacrylamide gel electrophoresis and then transferred onto PVDF membranes (Millipore, USA). 12–16% of total input cell lysates was loaded unless otherwise noted. Blocking was performed with 5% skim milk in TBS-T (TBS with 0.1% Tween-20) for 30 min at room temperature (RT). The membranes were probed with primary antibodies for 2 h at RT or overnight at 4 °C followed by secondary antibodies for 2 h at RT after washing 3 times with TBS-T for 8 min. The immunoblots were developed using an ECL reagent (Advansta, USA), according to the manufacturer’s protocol. The signals were developed by a film processor (Fujifilm FPM100A) and scanned with a commercial scanner. Adjustments of brightness, contrast or color balance were applied to the entire image.

Antibodies used include rabbit anti-PinX1 serum (prepared by our group), mouse monoclonal anti-GAPDH (Sigma, USA), mouse monoclonal anti-NPM (Abcam, USA), goat horseradish peroxidase-conjugated anti-mouse IgG (Bio-Rad, USA), and goat horseradish peroxidase-conjugated anti-rabbit IgG (Life Technologies, USA).

### Cell proliferation assay

Cell proliferation was determined using the CCK-8 Kit (Dojindo Laboratories, Kumamoto, Japan) according to manufacturer protocol. Briefly, the transfected HepG2 cells were seeded (5 × 10^3^ cells/well) on 6-well plates and cultured for 24–96 h. At 24, 48, 72 and 96 h, 10 μl of CCK-8 solution was added to each well and cultured for 2 h. Absorbance of the plate was measured at 450 nm with a microplate reader.

### Synchronization of HeLa Cells and Flow Cytometry

Cells were treated with 2 mM Hydroxyurea in culture medium with 10% FBS for 16–18 h for synchronization in G1/S phase boundary. Cells were washed once by warm PBS and then cultured in regular medium to release from hydroxyurea block. Cells were collected at different time points after release as indicated. For analysis of population of cells at each time point, harvested cells were washed once with PBS and then fixed with 1 ml ice cold 70% ethanol. Cells were fixed in 70% ethanol for overnight at − 20 °C. Fixed cells were collected and washed once with PBS. 1 ml PI DNA staining buffer (20 μg/ml Propidium Iodide, 10 μg/ml RNase in PBS) was added for resuspension and the cells were incubated at 37 °C in dark for 30 min. The cell cycle stages of the stained cells were analyzed by Flow cytometry (FACSCanto, BD).

### Immunofluorescence (IF)

HeLa cells were seeded and cultured on 13-mm circle cover glasses (Thermo-Menzel, Germany) in a 24-well plate. The cells were transfected accordingly (see above) if necessary. The cells were washed once with warm PBS and then fixed and permeabilized with paraformaldehyde for 10 min at 4 °C. The cells were washed thrice with PBS and incubated with PBS/5% Triton-X for 15 min. After washing twice with PBS, the cells were blocked in Blocking Buffer (5% FBS in PBS) at room temperature for 30 min or 4 °C overnight. Primary antibodies were added to the cover glass and incubated at room temperature for 2 h. The primary antibodies used were as follows: Mouse IgM Anti-hTERT 1:3000 in 5% FBS (2C4, Abcam, USA), Mouse monoclonal Anti-Coilin 1:500 in 5% FBS (ab87913, Abcam, USA), Mouse Anti-NPM 1:500 in 5% FBS (ab10530, Abcam, USA) and Rabbit Anti-PinX1 1:500 in 5% FBS (serum prepared by us). The primary antibodies were removed, and the cells were washed 3 times with PBS. Secondary antibodies were added to the cover glass and incubated at room temperature for 90 min.

The secondary antibodies used were: Cy2-conjugated AffiniPure Goat Anti-mouse IgM, μ Chain Specific 1:100 in 5% FBS (Jackson ImmunoResearch, USA), Dylight 594 AffiniPure Goat Anti-mouse IgG, Fc Fragment Specific 1:200 in 5% FBS (Jackson ImmunoResearch, USA), AlexaFluor 594 Goat anti-rabbit 1:500 in 5% FBS and AlexaFluor 488 Goat anti-mouse 1:500 in 5% FBS (Invitrogen, USA). The secondary antibodies were removed, and the nuclei were stained with 0.5 μg/ml 4′,6-diamidino-2-phenylindole (DAPI) for 1 min at room temperature. The cells were washed with PBS 3 times and then mounted on the glass slides with mounting medium (Dako, Agilent, USA).

### Microscopy

Immunofluorescence analysis was performed using an Olympus IX71 research inverted microscope with fluorescence observation. Images were acquired at 40× magnification by cooled CCD camera Olympus DP30BW. Images were merged by OLYMPUS MICRO software.

### Statistical analysis

Data are representative of at least three replicate experiments unless otherwise stated. Results are expressed as mean ± standard error of the mean (SEM) when appropriate. Correlational analysis of the immunofluorescence images was performed using ImageJ (ver. 1.48; National Institute of Health, USA) with the Colocalization Finder plugin (Laummonerie & Mutterer, Institut de Biologie Moleculaire des Plantes, France) and expressed as Pearson’s correlation coefficient (r).

## Results

### NPM and PinX1 work in the same telomere length regulation pathway

**Fig. 1:**
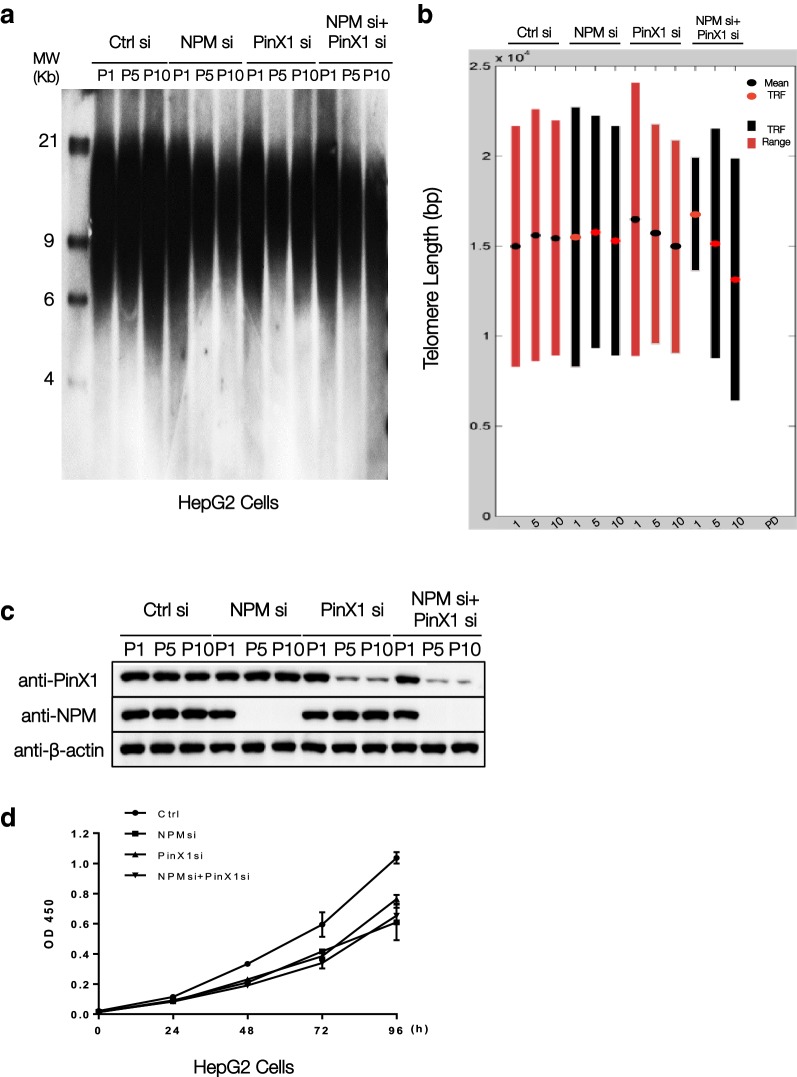
Long-term concurrent knockdown of PinX1 and NPM leads to substantial telomere length decrease in HepG2 cells. PinX1 and/or NPM siRNA were transfected to HepG2 cells every 9 days and the cells were reseeded every 3 days. **a** Southern blot of transfected HepG2 cells using digosin end-labeled (CCCTAA) oligonucleotide probe. **b** The standardized mean of the overall telomere length (Kb) for respective cell mass in 1, 5, and 10 PDs measured by Telotool. The dots indicate the average telomere lengths and the bars indicate the range values of telomere restriction fragments. **c** Western blots showing the expression profile of respective cell mass analyzed by anti-PinX1 and anti-NPM antibodies. GAPDH was used as loading control. **d** Cell proliferation of the transfected cells was determined with the CCK-8 Kit after cultured for 24-96 h

PinX1 is responsible for NPM loading onto hTERT [13]. To further characterize the relationship between PinX1/NPM interaction and telomerase activity, the long-term effect of PinX1/NPM down-regulation was observed in mammalian cells. si-RNA was transfected repeatedly to knock-down the endogenous protein in HepG2 cells. Cells were counted and reseeded every 3 days and the average telomere length of the cell line culture was measured by TRF assay for 1, 5, and 10 population doublings (PDs) (Fig. 1a, b). Western blots were used to show the down-regulation by siRNA (Fig. 1c). The level of cell proliferation was consistent across the long-term down-regulated cell cultures (Fig. 1d). It can be observed from the untreated control that the average telomere length of the cell is around 15 kb. Long-term down-regulation of NPM had minimal effect on the average telomere length 1 PD through 10 PDs. In 1 PD, an increase in telomere length was observed in PinX1 knock-down compared to the control, which reflected the inhibitory property of PinX1 on telomerase. At the same time, long-term PinX1 down-regulation also led to a decrease of average telomere length from 1 PD to 10 PDs. This suggested PinX1 has a positive effect on long-term telomere length maintenance and supported a duality of roles in telomere regulation. Meanwhile, concurrent PinX1 and NPM long-term knock-down led to a substantial decrease in average telomere length from 1 PD to 10 PDs that was greater than both singular NPM/PinX1 knock-downs. Interruption of endogenous NPM/PinX1 complex formation affects telomere-length maintenance efficacies. Considering PinX1 is responsible for mediating the hTERT association of NPM, this result supported NPM/PinX1 as a putative novel telomerase functional modulating unit, most likely by modulating cellular telomerase catalytic activity in accordance to to initial finding that PinX1 recruited NPM to hTERT for telomerase activation and subsequent telomere length maintenance.

### PinX1 and NPM co-localize in S phase

**Fig. 2:**
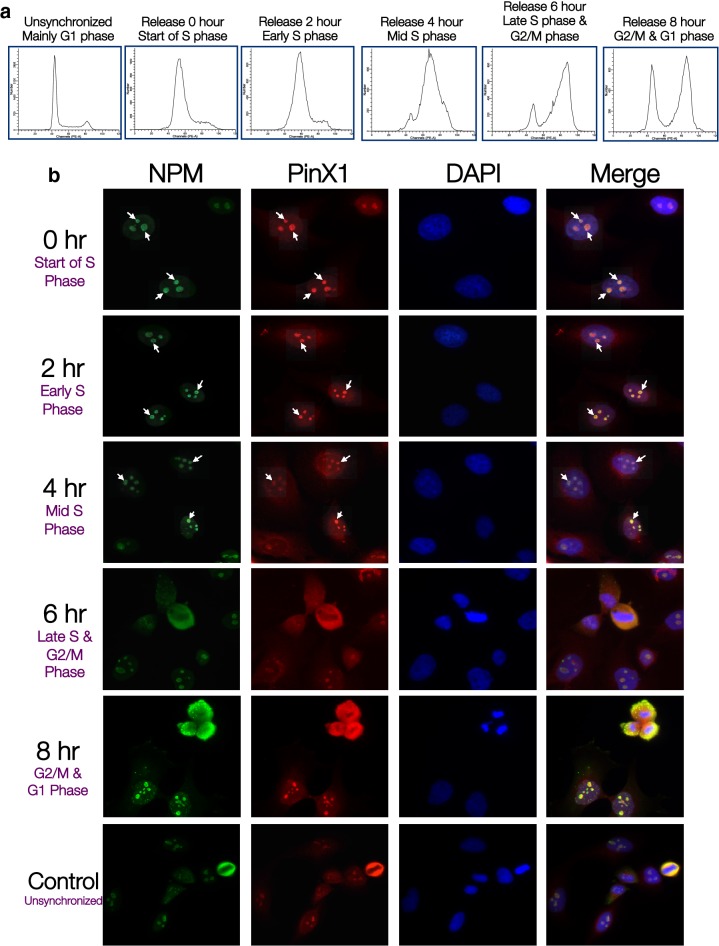
PinX1 and NPM co-localize during S Phase. HeLa cells were synchronized at G1/S boundary by 2 mM hydroxyurea and were fixed at indicated time points. **a** Flow cytometry analysis of PI stained cells showing cell cycle stages of population. **b** Immunofluorescence analysis showing localization of PinX1 and NPM at indicated stages of cell cycle after release from hydroxyurea block with anti-NPM (*green*) and anti-PinX1 (*red*). DAPI (*blue*) shows nucleus staining. The white arrows indicate co-localization between PinX1 and NPM

Since telomere elongation by telomerase occurs during S phase [23], the PinX1/NPM interaction and by extension the hTERT/PinX1/NPM complex formation should also occur during the time of telomerase activation for PinX1/NPM to play a part in telomere length homeostasis. To examine this, endogenous immunofluorescence experiment was performed in G1/S boundary synchronized HeLa cells. The cells were fixed at indicated time points and the cell cycle stages were analyzed by flow cytometer (Fig. 2a). Hydroxyurea block was released 16–18 h after treatment and the proteins were stained by corresponding antibodies. Intensity correlation of co-localizing signals was expressed as Pearson’s correlation coefficient and presented in Additional file 1: Table S1. Co-localization between PinX1/NPM was observed in the majority of the cell population in focused nucleolar loci after the release of the block to 6 h, which corresponded to the start of S phase to the late-S phase. After the late-S phase, PinX1/NPM diffused from nucleolar loci to scatter within nucleoplasm (Fig. 2b). This suggested that PinX1/NPM interaction peaked during early- to mid-S Phase and continued until the beginning of the late-S phase.

### NPM and hTERT co-localize in early-S phase

**Fig. 3:**
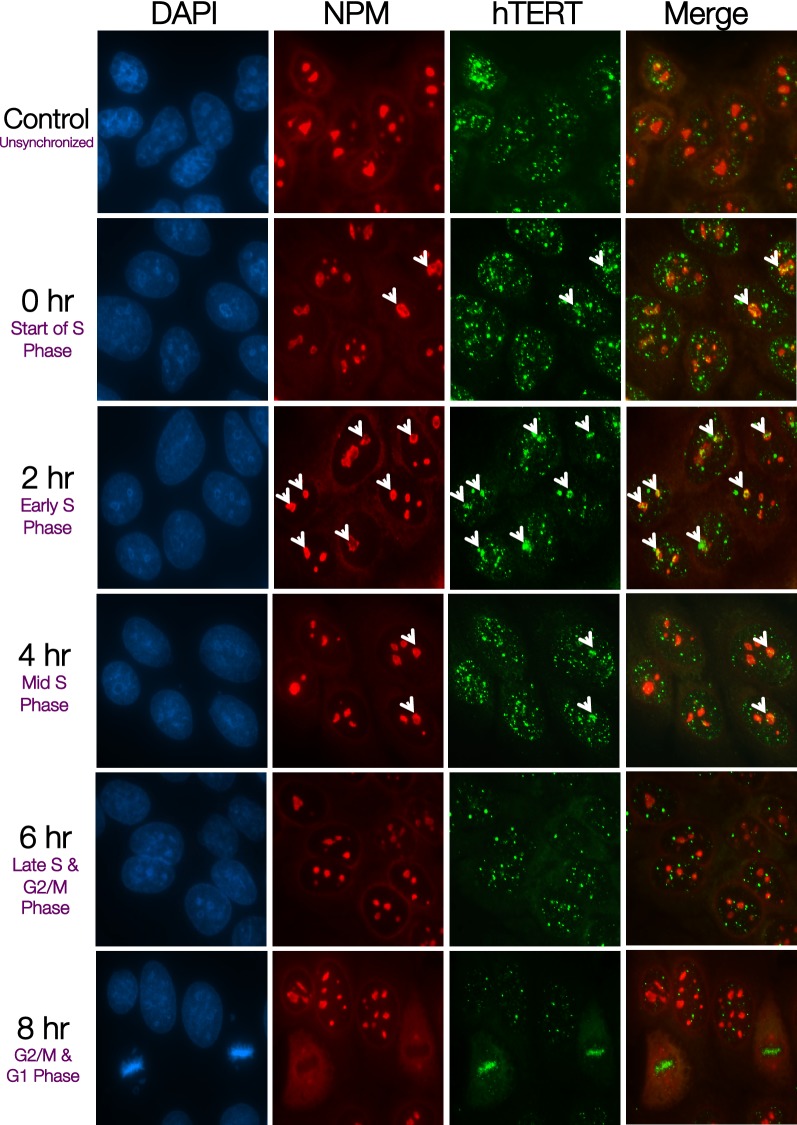
NPM and hTERT co-localize during early-S Phase. HeLa cells were synchronized at G1/S boundary by 2 mM hydroxyurea and were fixed at indicated time points. Immunofluorescence analysis showing localization of NPM and hTERT at indicated stages of cell cycle after release from hydroxyurea block with anti-NPM (*red*) and anti-hTERT (*green*). DAPI (*blue*) shows nucleus staining. The white arrows indicate co-localization between NPM and hTERT

Similarly, hTERT was found to scatter within the nucleus in the unsynchronized population (mainly in G1 phase) and the synchronized cells at the G1/S boundary. In early-S phase when telomerase catalysis occurs, majority cell population showed translocation of hTERT from scattered nucleoplasm loci to the nucleolus and formation of clusters at the location of NPM (Fig. 3). From mid-S phase onwards, fewer cells showed hTERT nucleolar localization with NPM and hTERT retained its scattered loci within the nucleus, partially translocated to telomere speckles as reported previously [20]. During the mitotic phase, hTERT localized with the condensed chromosomes while NPM demonstrated nucleoplasm scattered pattern. This showed the dynamic movement of hTERT and NPM during cell cycle progression—NPM and hTERT were found to co-localize the most during the early-S phase (Additional file 1: Table S2). Taken together, the association of the NPM/PinX1/hTERT complex appeared to peak during the early-S phase. This indicated that NPM is likely to associate with hTERT and attenuate PinX1 inhibition at early-S phase, and thus facilitating the activation of telomerase.

### PinX1 facilitates NPM loading to hTERT during early-S phase

**Fig. 4:**
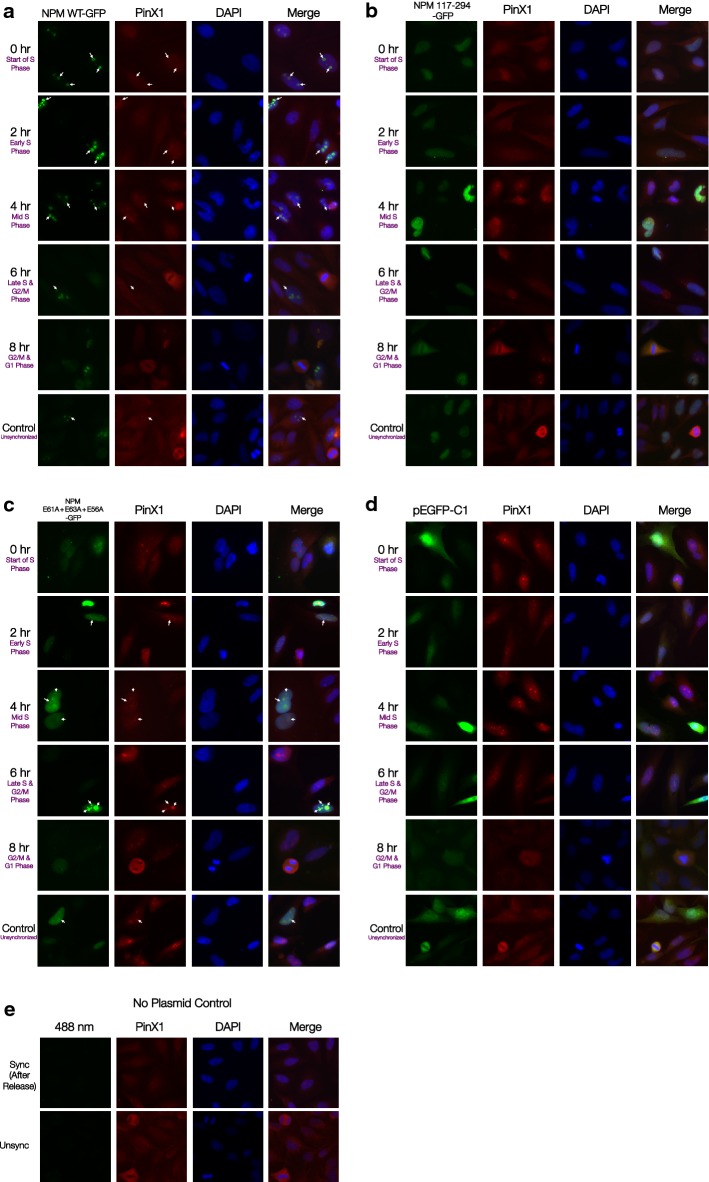
PinX1 is critical in loading NPM to hTERT during early-S Phase. HeLa cells were transfected with respective plasmids for 24 h and synchronized at G1/S boundary by 2 mM hydroxyurea and were fixed at indicated time points. Immunofluorescence analysis showing localization of **a** GFP-tagged wildtype NPM and PinX1; **b** GFP-tagged NPM aa117-294 and PinX1; **c** GFP-tagged NPM E61A + E63A + E56A mutant and PinX1; **d** pEGFP-C1 vector and PinX1; **e** Synchronized and unsynchronized non-transfection controls, at indicated stages of cell cycle after release from hydroxyurea block with GFP-tagged signals (*green*) and anti-PinX1 (*red*). DAPI (*blue*) shows nucleus staining. The white arrows indicate co-localization between GFP-tagged proteins and PinX1 where appropriate

To further investigate the significance of PinX1 association to NPM in the context of telomerase catalysis, immunofluorescence studies were performed with GFP-tagged wild-type and mutant NPM. In our previous work, we have revealed a significant decrease in PinX1 association with NPM E61A + E63A + E56A mutant compared to wildtype NPM, and NPM’s ability to interact with PinX1 directly correlated with its ability to regulate telomerase activity [13]. Here, we observed scattered NPM loci throughout S phase in truncation mutant NPM aa117-294 (with a complete loss of PinX1 interaction region) with significantly reduced PinX1 co-localization (Fig. 4b; Additional file 1: Table S3). At the same time, NPM WT-GFP and PinX1 demonstrated co-localization pattern throughout S phase that peaked in early- to mid-S phase (Fig. 4a; Additional file 1: Table S3), which corresponded to the co-localization pattern of endogenous NPM and PinX1.

However, with NPM E61A + E63A + E56A mutant, scattered NPM loci was observed after the release of hydroxyurea block. The NPM E61A + E63A + E56A mutant did not demonstrate complete abolishment of PinX1 co-localization akin to the NPM aa117-294 pattern. Co-localization between this NPM mutant and PinX1 peaked during mid- to late-S phase (Fig. 4c; Additional file 1: Table S3). The delay in recruitment by PinX1 was consistent with the significant reduction in the ability of NPM E61A + E63A + E56A mutant to associate with PinX1. Oligomerization between endogenous wildtype NPM and the mutant NPM could also take place [24] and caused the GFP signals to retain co-localization pattern in later stages of the cell cycle. Still, this result suggested that the ability to associate with PinX1 is critical in the nucleolar localization of NPM during the S phase of the cell cycle. Since NPM binding to PinX1 is required for NPM loading to hTERT and PinX1 is critical in localizing NPM in nucleolus during the early-S phase, PinX1 is likely to mediate NPM loading to telomerase during telomere catalysis and this confirms the PinX1/NPM interaction is involved in a telomerase activation pathway.

### PinX1/NPM does not co-localize in Cajal Bodies at the start of S phase

To support the hypothesis that the PinX1/NPM interaction is likely to regulate telomerase catalysis through the formation of NPM/PinX1/hTERT complex during the early-S phase of the cell cycle, the localization of PinX1/NPM relative to Cajal bodies (CBs) was investigated.

**Fig. 5:**
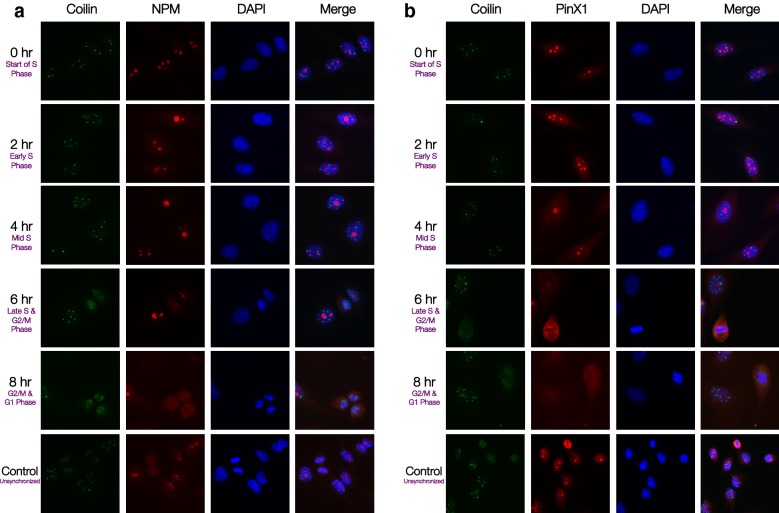
PinX1/NPM does not localize at Cajal Bodies at the start of S Phase. HeLa cells were synchronized at G1/S boundary by 2 mM hydroxyurea and were fixed at indicated time points. Immunofluorescence analysis showing localization of **a** NPM and coilin; and **b** PinX1 and coilin, at indicated stages of cell cycle after release from hydroxyurea block with anti-coilin (*green*) and anti-NPM (*red*) in (A); anti-PinX1 (*red*) in (B), and DAPI (*blue*) shows nucleus staining

Coilin is the integral component and protein marker for CBs. Hence, the localization of coilin would reveal the subnuclear domain of CBs. The localization patterns between coilin and PinX1 and NPM respectively were studied by immunofluorescence in HeLa cells. Consistent with previous findings [19, 25], coilin condensed in multiple clusters in the peri-nucleolar space outside the nucleolus in asynchronized HeLa cells. In synchronized cells, neither PinX1 nor NPM co-localized with coilin throughout S phase (Additional file 1: Table S4). At the start of S phase (after the release of hydroxyurea block), coilin demonstrated a highly concentrated pattern and the least number of nucleoplasmic clusters. In the early- to mid-S phase, the coilin clusters decreased in size and translocated to scattered loci. Coilin signals began to overlap with PinX1 and NPM signals at the later stages of S phase (Fig. 5). As cells entered G2/M and G1 phase in the cell cycle, chromatin condensed as indicated by DAPI staining and coilin dispersed within nucleoplasm as CBs disassemble in M phase. The result indicated that PinX1/NPM did not localize at CBs at the start of S phase and were further away from the CBs at the start than at the end of S phase. Taken together, this suggested that the PinX1/NPM interaction is more likely to regulate telomerase activation during catalysis, rather than during subnuclear events such as telomerase assembly prior to the start of S phase.

## Discussion

Since its discovery as a potent telomerase inhibitor in 2001 [6], PinX1 had been revealed to be involved in tumorigenesis and tumor progression, and evidence suggested that its functions could be tumor-type-specific [26]. PinX1 has emerged as a potential tumor marker. For instance, PinX1 expression has recently been proposed as a prognostic factor for human non-small cell lung cancer [11]. Meanwhile, it is intriguing that PinX1 is also involved in loading telomerase to telomere in addition to its telomerase activity inhibition property. Previously, PinX1 was revealed to be responsible for loading its interacting partner nucleophosmin (NPM) to hTERT, the catalytic subunit of telomerase, and NPM can partially reverse the inhibition to telomerase activity by PinX1 [13]. However, the mode and timing of the PinX1/NPM interaction in the cell cycle in correspondence to telomere catalysis were not characterized. We hypothesized that PinX1 recruits the positive telomerase regulator NPM to hTERT to activate telomerase and facilitate catalysis. Here, the proposed novel PinX1/NPM telomerase modulating unit was investigated.

Firstly, our long-term down-regulation results suggested that PinX1/NPM is a telomerase functional modulating unit in the regulation of telomerase activity and telomere length maintenance. PinX1 involvement in a telomerase-independent mechanism that led to telomere dysfunction had been suggested in osteosarcoma cells [27]. Here, the expression of the level of NPM in HepG2 cells had been found to have minimal effect on telomere length consistently from the short-term (1 PD) to the long-term (10 PD). And while the silencing of PinX1 was found to increase telomere length in the short-term, it compromised telomere length maintenance slightly in the long-term. This reflected PinX1′s telomerase inhibitory property as well as its telomere maintenance property reported in previous findings [6, 11]. Furthermore, the concurrent knock-down of PinX1 and NPM led to an increase in telomere length in the short-term similar to that with PinX1 singular knock-down, but a substantial decrease in the long-term that was greater than that with PinX1 singular knock-down. This suggested a synergism between PinX1 and NPM in regulating telomere. Taking into account the attenuation ability of NPM against PinX1 in recovering reduced telomerase activity and that NPM requires PinX1 to load onto hTERT, this data supported PinX1/NPM as a closely functioning unit in telomerase/telomere regulation.

Next, our immunofluorescence studies revealed the localization of PinX1, NPM, and hTERT during the progression of the cell cycle. Telomerase is highly activated in S phase, which is when telomere extension and DNA replication occur [23]. NPM predominantly localizes in nucleolus and hTERT scatters within nucleoplasm until telomerase catalysis. Endogenous PinX1/NPM association was consistent throughout S phase, while NPM and hTERT co-localization peaked in early-S phase. NPM/PinX1 undergoes a dynamic regulated subcellular distribution by concentrating foci from early-to mid-S phase and dispersing into diffused distribution entering G2/M phase. Hence, the formation of NPM/PinX1/hTERT should peak during the early-S phase when telomere catalysis is highly activated. Moreover, by observing the localization pattern of non-PinX1-interacting NPM E61A + E63A + E56A mutant, we also demonstrated that the interaction with PinX1 is critical for NPM translocation to nucleolus from nucleoplasm foci. This correlates with the duality of PinX1 in telomerase activation—as a telomerase activity inhibitor and as a linker for positive telomerase regulatory proteins—and supports the notion that PinX1/NPM exists as a telomerase modulating unit.

To further support this, the localization of PinX1/NPM relative to Cajal Bodies (CBs) marker coilin was observed. CBs are subnuclear organelles near nucleolus where telomerase is recruited, and the sites for hTR accumulation and telomerase complex assembly in telomerase active cells [28]. Meanwhile, coilin is an integral component of CB and widely regarded as its protein marker [19, 29]. It has also been reported that coilin is involved in hTR processing [30]. Overall, the immunofluorescence analysis showed that the PinX1/NPM interaction did not localize at CBs at the start of S phase. This decreases the likelihood of the PinX1/NPM interaction participating in the nuclear events at CBs prior to telomerase catalysis in S phase. Thus, the proposed telomerase modulating unit likely activates telomerase through NPM, which is mediated by PinX1, during catalysis.

As mentioned, telomere extension only occurs in S-phase in the cell cycle and the telomerase remains in its inactive state in the rest of the time [23]. At the same time, the expression level of telomerase inhibitor PinX1 remains constant throughout the cell cycle [31]. This suggests the existence of a protein switch that allows telomerase activation and inactivation in the PinX1 regulation pathway during the cell cycle. The results presented here strongly support the existence of a functional modulating unit that regulates telomerase catalysis involving PinX1/NPM. Meanwhile, a catalytic telomerase CBs assembly regulation pathway constituted by NPM/PinX1/hTERT and subsequent coilin-guiding telomerase translocation to telomere are also possible.

## Conclusion

We propose PinX1/NPM as a telomerase modulating unit that regulates telomerase catalysis during S phase of the cell cycle. The concurrent long-term down-regulation of PinX1 and NPM leads to a substantial decrease in telomere length, suggesting the two proteins work closely to modulate telomerase function. PinX1, NPM, and hTERT associate as a complex, of which formation peaks in early-S phase. Furthermore, PinX1 is critical in localizing positive telomerase regulator NPM to the nucleolus and acts as a linker for its association with hTERT, which allows NPM to activate telomerase.

## Additional files

**Additional file 1.** Correlation Analysis for Immunofluorescence Images. Degree of colocalization between proteins expressed as Pearson’s correlation coefficient of the two immunofluorescence signals.

## Abbreviations

NPM: nucleophosmin
hTR: telomerase RNA
hTERT: telomerase reverse transcriptase
CBs: Cajal bodies

## Availability of data and materials

All data generated or analyzed during this study are included in this published article.
