# Supplementary material for: The PinX1/NPM interaction associates with hTERT in early-S phase and facilitates telomerase activation
Source: Cell Biosci. 2019 Jun 13;9:47. doi: 10.1186/s13578-019-0306-y (PMC6567508; doi:10.1186/s13578-019-0306-y)
Supplement: Supplementary file 1 — Additional file 1. Correlation Analysis for Immunofluorescence Images. Degree of colocalization between proteins expressed as Pearson’s correlation coefficient of the two immunofluorescence signals. [file 13578_2019_306_MOESM1_ESM.pdf]

Correlation Analysis for Immunofluorescence Images

Table 1. Correlation analysis of co-localization between PinX1 and NPM signals (Fig. 2)

Time after release of hydroxyurea block	Pearson's correlation coefficient
0 hr	0.824 ± 0.003
2 hr	0.802 ± 0.070
4 hr	0.688 ± 0.107
6 hr	0.645 ± 0.109
8 hr	0.466 ± 0.003
Control	0.667 ± 0.130

Table 2. Correlation analysis of co-localization between NPM and hTERT signals (Fig. 3)

Time after release of hydroxyurea block	Pearson's correlation coefficient
0 hr	0.307 ± 0.047
2 hr	0.524 ± 0.090
4 hr	0.429 ± 0.030
6 hr	0.325 ± 0.096
8 hr	0.238 ± 0.042
Control	0.244 ± 0.020

Table 3. Correlation analysis of co-localization between NPM variants and PinX1 signals (Fig. 4)

Time after release of hydroxyurea block	Pearson's correlation coefficient			
	NPM WT	NPM 117-294	NPM E61A + E63A + E56A	GFP
0 hr	0.499 ± 0.074	0.247 ± 0.018	0.281 ± 0.064	0.190 ± 0.052
2 hr	0.542 ± 0.069	0.347 ± 0.131	0.367 ± 0.078	0.211 ± 0.081
4 hr	0.426 ± 0.068	0.334 ± 0.137	0.636 ± 0.122	0.200 ± 0.007
6 hr	0.265 ± 0.047	0.349 ± 0.035	0.662 ± 0.056	0.243 ± 0.009
8 hr	0.205 ± 0.019	0.431 ± 0.068	0.281 ± 0.054	0.275 ± 0.048
Control	0.569 ± 0.042	0.302 ± 0.046	0.317 ± 0.175	0.244 ± 0.102

Table 4. Correlation analysis of co-localization of coilin/PinX1 and coilin/NPM signals (Fig. 5)

**Time after release of hydroxyurea block**	**Pearson's correlation coefficient**	
	Coilin/PinX1	Coilin/NPM
0 hr	-0.005 ± 0.049	-0.013 ± 0.037
2 hr	0.065 ± 0.037	-0.0004 ± 0.027
4 hr	0.032 ± 0.020	0.035 ± 0.054
6 hr	0.159 ± 0.089	0.083 ± 0.026
8 hr	0.223 ± 0.017	0.183 ± 0.054
Control	0.070 ± 0.027	0.046 ± 0.043
